# Treatment of Acute Pneumonia

**Published:** 1866-08

**Authors:** 


### Treatment of Acute Pneumonia.

In a clinical lecture, at the Mater Misericordiæ Hospital, Dr. Hughes gave, as his opinion, that no peculiar or specific treatment is applicable to all cases of acute pneumonia, but that the local inflammation in each case subsides concurrently with the fever. He exemplifies his views by two cases in point, in both of which the local mischief was pretty much the same, but the accompanying fever was very different, in the one being of a high grade, with pulse 130, respiration 40, and tongue foul, white furred and thickly coated. In the other the fever was of a lower grade; pulse 80, small and weak; respiration 30, tongue coated with a white, *blankety* fur, and red at the tip. The first case was treated with tartar emetic, the second with moderate stimulants, local counter-irritation being applied in both cases. The recovery was very rapid in both instances. The medical literature of the day abounds in recommendations from various authorities as to the treatment of pneumonia, each succeeding one warning his readers against the mistakes of his predecessors and urging the adoption of his special plan. Dr. Hughes sees in this difference of opinion a proof that if we are desirous of obtaining a sure guide to the successful treatment of pneumonia, and other kindred states of the system, we must study the type of the accompanying fever and adapt our treatment to it, instead of vainly endeavoring to make one plan of treatment fit all cases. He thinks that if the same plan of treatment had been pursued in these two cases, there are good grounds for believing that the results would not have been so favorable in both.—*N. Y. Medical Journal.*

